# Gene panel for the diagnosis of epidermolysis bullosa: proposal for a viable and efficient approach^☆^^☆☆^

**DOI:** 10.1016/j.abd.2020.05.015

**Published:** 2021-02-02

**Authors:** Luiza Monteavaro Mariath, Ana Elisa Kiszewski, Jeanine Aparecida Frantz, Marina Siebert, Ursula Matte, Lavínia Schuler-Faccini

**Affiliations:** a Graduate Program in Genetics and Molecular Biology, Universidade Federal do Rio Grande do Sul, Porto Alegre, RS, Brazil; b Dermatology Section, Universidade Federal de Ciências da Saúde de Porto Alegre, Porto Alegre, RS, Brazil; c Dermatological Pediatrics Section, Hospital da Criança Santo Antônio, Irmandade da Santa Casa de Misericórdia de Porto Alegre, Porto Alegre, RS, Brazil; d School of Medicine, Universidade Regional de Blumenau, Blumenau, SC, Brazil; e DEBRA Brasil, Blumenau, SC, Brazil; f Experimental Research Center, Hospital de Clínicas de Porto Alegre, Porto Alegre, RS, Brazil; g Instituto Nacional de Ciência e Tecnologia de Genética Médica Populacional (INaGeMP), Porto Alegre, RS, Brazil

**Keywords:** Genetic counseling, Diagnosis, Epidermolysis bullosa, Genetic testing

## Abstract

**Background:**

Epidermolysis bullosa is characterized by cutaneous fragility and blistering. Historically, diagnosis is achieved by immunofluorescence mapping or transmission electron microscopy, both involving biopsy procedures. Genetic analysis, especially through next-generation sequencing, is an important tool for the diagnosis of this disease. In Brazil, access to diagnostic methods is limited, and consequently, most patients do not have an accurate diagnosis. Diagnosis allows the indication of prognosis and genetic counselling of the patient.

**Objectives:**

To evaluate the cost-effectiveness of a gene panel compared to immunofluorescence mapping and transmission electron microscopy by analyzing its benefits, limitations, and economic aspects.

**Methods:**

The gene panel included the 11 main genes associated with epidermolysis bullosa. The techniques were compared, assessing the average cost, advantages, and limitations, through a price survey and literature review.

**Results:**

Both immunofluorescence mapping and transmission electron microscopy require skin biopsy, are dependent on the investigator’s expertise, and are subject to frequent inconclusive results. The gene panel is effective for the conclusive diagnosis of epidermolysis bullosa, presents high efficiency and accuracy, is economically feasible, and excludes the need for biopsy. The gene panel allows for prognosis, prenatal genetic diagnosis, and genetic counseling.

**Study limitations:**

It was not possible to find laboratories that perform transmission electron microscopy for epidermolysis bullosa diagnosis in Brazil.

**Conclusion:**

This study supports the gene panel as the first-choice method for epidermolysis bullosa diagnosis.

## Introduction

Epidermolysis bullosa (EB) is a heterogeneous group of genetic skin diseases characterized by skin fragility, with the formation of blisters, erosions, and scars in response to minimal mechanical trauma.^1^ EB is classified into four main types, according to the layer of the skin in which the formation of blister occurs: EB simplex (EBS; intraepidermal), junctional EB (JEB; within the lamina lucida of the basement membrane), dystrophic EB (DEB; below the basement membrane), and Kindler EB (mixed skin cleavage pattern).^1^, ^2^ The classification of EB takes into account the clinical characteristics, the distribution of blisters (localized or generalized), and the severity of cutaneous and extracutaneous signs.^2^ Pathogenic variants in at least 16 genes, which encode proteins with structural functions, are responsible for the genetic and allele heterogeneity of EB.^2^, ^3^

In the United States, the incidence and prevalence of EB were estimated at 19.57 and 11.07 per million individuals, respectively.^4^ In Brazil, there are no epidemiological studies on EB. DEBRA-Brasil, in an effort to register Brazilian EB patients, has a record of more than 900 cases in the country (unpublished data). It is estimated that the number of Brazilian patients is even higher, given the underdiagnosis of the disease, especially in less severe cases, errors in diagnosis, and premature death in severe cases, combined with the difficulty in accessing the main health centers.

The diagnosis of EB in specialized centers worldwide is carried out mainly by clinical examination followed by the technique of direct immunofluorescence on tissue (known as immunofluorescence mapping or immunomapping) and/or transmission electron microscopy, performed in biopsy samples of skin.^5^ When accessible, genetic testing is also an important tool for the diagnosis of EB. The classification of the type of EB contributes to the prognostic evaluation of the patient and is important for genetic counseling of the family.

Immunofluorescence mapping (IFM) is based on the use of specific antibodies that enable the identification of the non-functional protein and the layer of the skin where cleavage occurs in the most severe types of EB.^5^, ^6^, ^7^ However, the information is dependent on the quality and number of antibodies used and inconclusive results can occur due to different factors. Firstly, cleavages can be formed in the skin sample during storage and processing, leading to inaccurate results.^3^, ^7^ In addition, the cleavage may not be present and, consequently, it is not possible to classify EB in these cases.^7^ Finally, the interpretation of the results depends on the researcher's knowledge and expertise, which requires a specialized team in order to reach the correct diagnosis.^3^, ^5^

Transmission electron microscopy (TEM) also allows the diagnosis of EB through the determination of the skin layer in which the cleavage occurs. As a differential, the technique also allows the observation and semiquantitative evaluation of structures that are altered in specific subtypes of EB (keratin filaments, desmosomes, hemidesmosomes, filaments, and anchoring fibrils).^5^ However, it is a high-cost method and is difficult to process and analyze, thus requiring highly qualified laboratories with appropriate expertise.^5^ In addition, TEM results may be non-informative, due to the absence of skin cleavage, non-specific alterations, or very subtle changes in the adhesion structures. Artifacts can also occur due to processing and biopsy techniques.^3^ As few laboratories are able to perform the technique, TEM is rarely the first choice for the diagnosis of EB.

Until recently, genetic diagnosis of patients with EB was only possible through Sanger sequencing of candidate genes, defined after the completion of IFM and/or TEM, a laborious and costly strategy.^1^ Recently, next-generation sequencing (NGS) has emerged as a useful tool for the identification of genetic variants, and studies in EB have demonstrated its high diagnostic sensitivity.^8^, ^9^, ^10^, ^11^, ^12^

In Brazil, access to EB diagnostic methods is available to only a small portion of patients. The first reason is due to the technical difficulties involved, which requires specialists for analysis. Although this difficulty is common to all who perform the techniques, it is even more striking in a country such as Brazil, due to the shortage of experienced analysts. The second reason is related to the insufficiency of specialized health centers in Brazil and the unavailability of tests in the public healthcare system. Consequently, most Brazilian patients with the disease do not have a precise and conclusive diagnosis of the type of EB, either due to technical difficulties, which lead to inconclusive results, or lack of access to these methods in the country.

In view of the difficulty of diagnosing the disease in Brazil, the authors propose a specific gene panel for EB through NGS technology. In a previous study, the authors presented the new and recurring variants identified in Brazilian patients through the gene panel.^13^ The aim of the present study is to evaluate the cost-effectiveness of the gene panel in comparison with the historically employed tests, discussing the main advantages and limitations of each method, as well as the economic aspects.

## Methods

### Ethical aspects

The present study was approved by the Research Ethics Committee of the Universidade Federal do Rio Grande do Sul (project 31608) and by Plataforma Brasil (CAAE 2,481,885).

### EB gene panel

The NGS gene panel was designed to be specific for EB, including the vast majority of cases of the disease at a viable cost for the Brazilian reality. Thus, the 11 main genes associated with the four types of EB were included in the panel (*KRT5*, *KRT14*, *PLEC*, *TGM5*, *LAMA3*, *LAMB3*, *LAMC2*, *COL17A1*, *ITGB4*, *COL7A1*, and *FERMT1*). The 62.72-kb and 574-amplicon panel was designed using the AmpliSeq Designer tool (Thermo Fisher Scientific – United States). Ion Torrent technology (Thermo Fisher Scientific – United States) was used for sequencing. Information about the running parameters, data analysis, investigation, classification, and details of the identified variants were described in a previous study.^13^

### Evaluation of diagnostic methods

In order to obtain an estimate of the amount to be invested in the execution of IFM, TEM, and gene panel sequencing, a price survey was carried out in Brazilian hospitals/laboratories that perform each of the methods. For the TEM technique, a survey was carried out in laboratories in the United States, due to the lack of Brazilian laboratories that perform the method for the diagnosis of EB. Since the cost of gene panel sequencing is dependent on the number of samples to be analyzed, for calculation purposes, the price estimate took into account a number of 20 samples. The characteristics of each method, as well as their inherent advantages and disadvantages, have been reviewed in the specialized literature.

## Results

The positive and negative aspects of the techniques used for the diagnosis of EB are compiled in Table 1. As demonstrated in a previous study,^13^ the efficiency of the developed gene panel was 94.3% (in 82 of the 87 index cases studied, pathogenic variants were identified). Although IFM and TEM have relative diagnostic accuracy, only the gene panel allows a complete and definitive analysis, with the identification of the causal genetic alteration. Moreover, one of the main benefits of the gene panel sequence is the lack of need for a biopsy (particularly harmful to these patients with sensitive skin), which is essential in the other two methods.

**Table 1:** Comparison between the techniques used for the diagnosis of epidermolysis bullosa.

	Immunofluorescence mapping	Transmission electronic microscopy	Gene panel by NGS
Number of laboratories	Main type of EB and the likely altered protein	Analysis of specific structures of EB subtypes	Identification of the genetic alteration
	Diagnostic accuracy	Diagnostic accuracy	Determination of EB type and inheritance pattern
	Fast execution		
	Prognostic value		No biopsy needed

			High efficiency (94.3%)^c^
			Diagnostic accuracy^d^
			Informative and conclusive for most cases
			Enables prenatal and pre-implantation genetic diagnosis
			Sample transportation easiness
**Disadvantages**	Biopsy	Biopsy	High price
	Dependent on the researcher's experience	High price	Expert analysis centers needed
	It is not possible to accurately distinguish all subtypes of EB	Few centers specialized in the technique	Experience required for interpretation and classification of variants
	Inconclusive results are common	Expertise required for correct analysis	
	Possible technical artifacts	Possible technical artifacts	
	No information on genetic alteration		
**Cost per patient**^a^	R$ 500.00	Not performed in Brazil^b^ (in the United States: US$ 980.00)	R$ 800.00

a Costs are based on the mean estimate of prices surveyed in the market.

b To the best of the authors’ knowledge, according to the research conducted in this study.

c Efficiency identified in a study with the proposed gene panel.^13^

d It is important to note the difference between "efficiency" and "accuracy." Efficiency refers to the proportion of solved cases (that is, cases with the identified pathogenic mutation) in relation to the total number of patients analyzed. The term "accuracy", in turn, refers to the precision (correctness) of the results.

The amount to be invested in the diagnosis of EB through the gene panel was estimated at R$ 800.00 per sample, taking into account a total of 20 samples analyzed. The mean price of IFM in the Brazilian market is R$ 500.00. After extensive research in different Brazilian reference centers, no laboratory was found to perform TEM for the diagnosis of EB in the country. For estimation purposes, a survey of the price of the technique was carried out in laboratories in the United States, which indicated a mean price of US$ 980.00 (R$ 1.00 = US$ 0.25).

**Figure 1:**
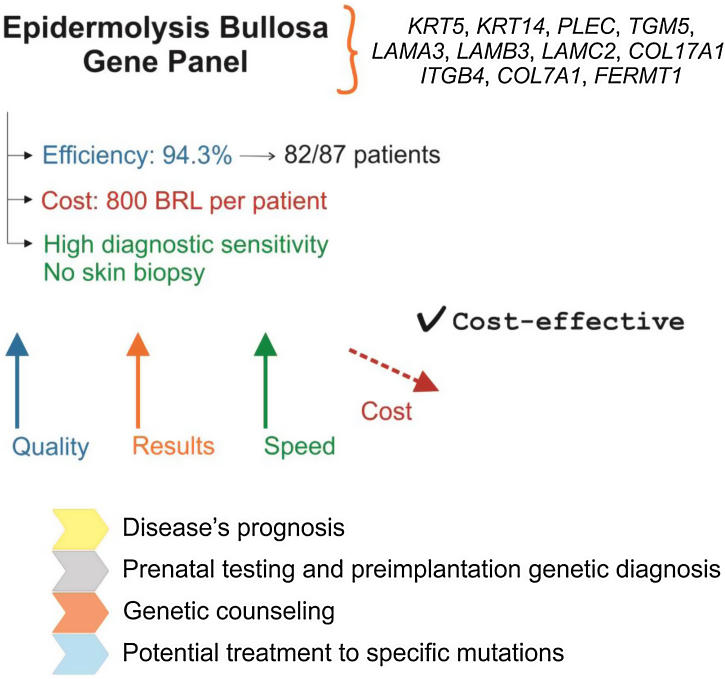
Gene panel as the first choice for the diagnosis of EB: main characteristics. Gene panel has high efficiency and precision, a price comparable to other EB diagnostic methods, and eliminates the need for skin biopsy. The cost of NGS technology has been decreasing, which indicates that the gene panel should become more accessible in the coming years. Identification of the genetic alteration allows establishing disease prognosis, prenatal and pre-implantation genetic diagnosis, has implications for genetic counseling, and is important for the development of future therapies for EB.

Fig. 1 presents a summary of the main characteristics, advantages, and implications of using the EB gene panel for the diagnosis of the disease.

## Discussion

In the present study, the authors present the advantages and disadvantages of diagnostic methods for epidermolysis bullosa and an assessment of the potential of the gene panel as the first choice for diagnosing the disease in Brazil.

### Gene panel vs. IFM and TEM: effectiveness, immediate benefits, and potential future

IFM and TEM are, historically, the most used methods for the diagnosis of EB and allow the determination of the level of cleavage in the skin and the consequent classification of the EB subtype.^5^ Although they have diagnostic accuracy for the main types of EB, these methods do not indicate the mode of genetic inheritance and depend on experience for analysis, being subject to frequent inaccurate or inconclusive results (Table 1).^3^, ^14^ TEM, in particular, is a very laborious technique that requires notable technical knowledge and is therefore very little used worldwide. The identification of the associated pathogenic variant is the most definitive and precise criterion for the diagnosis of EB. The sequencing of candidate genes by the Sanger method is, therefore, performed in specialized centers, after IFM and/or TEM.^5^, ^14^ Although informative in most cases, the combination of IFM, TEM, and Sanger sequencing methods fails in approximately 15% of cases, either due to uncertainty in the clinical examination or due to intrinsic limitations of the techniques.^8^ Moreover, this combined approach can be costly and requires a great deal of work, since several of the genes associated with EB contain multiple exons, such as *COL7A1* (118 exons) and *COL17A1* (56 exons).^8^

Unlike Sanger sequencing, the NGS gene panel allows the simultaneous sequencing of a large number of genes with a known association with the disease,^1^ waiving the need of previous determination of candidate genes. Genetic analysis by gene panel is fast, highly accurate, and conclusive for the diagnosis of EB, without requiring previous tests such as IFM and TEM (Table 1).

The IFM and TEM methods require skin biopsy, usually performed on a recent intact blister. This examination is in itself highly invasive, but in patients with EB, who have frail skin, it becomes even more aggressive. Furthermore, the need for repeated biopsy due to inconclusive results is not uncommon. Since NGS analyses do not require, can be performed on a saliva sample, and provide diagnosis with speed and high precision, some authors have advocated rethinking current methods for assessing patients with EB.^15^ The identification of causal variants associated with EB allows the indication of the prognosis of the disease, prenatal and pre-implantation genetic diagnosis, and precise genetic counseling.^3^

Although NGS sequencing is a powerful tool for genetic diagnosis, some limitations must be considered. The main challenge concerns bioinformatics analyses, which are essential for the identification of genetic variants and, even more important, for indicating their causality.^8^ In this sense, the interpretation of variants of uncertain meaning can be quite difficult, causing dilemmas for clinicians and uncertainties on how to inform patients and provide appropriate genetic counseling. In addition, large rearrangements and repetitive sequences are complex to analyze by NGS and, therefore, are often not correctly detected. Thus, it is important to note that, although highly effective, NGS technology cannot solve all cases of genetic diseases.

The gene panel developed by the present group showed high efficiency (94.3%) in the studied sample, when compared with the results obtained by other researchers who used panels with a larger number of genes.^10^, ^11^, ^12^ The choice of the 11 main genes associated with EB was made in order to establish an economically viable methodology, although other genes were also reported in the literature.^2^, ^3^ Although the panel developed fails to diagnose rare cases associated with other genes (such as STD, EXPH5, KLHL24, CD151, ITGA3, and ITGA6), the efficiency obtained here demonstrates that even a smaller gene panel can be of remarkable relevance for the diagnosis of EB. It should be noted that the NGS methodology does not allow for an adequate identification of large genetic rearrangements, which presupposes that even a panel including all the genes already associated with EB does not guarantee 100% efficiency.

Aside from the immediate benefits, genetic analysis can be an essential tool for the development and employment of EB treatment. Although there is currently no treatment, recent preclinical studies have suggested potential therapies for EB, many of them dependent on the associated pathogenic variant.^16^, ^17^, ^18^, ^19^, ^20^ The advancement in genetic diagnosis through NGS technology, together with the development of new molecular therapies, promises to bring significant benefits to this group of patients.^21^ Although it is not a current reality, it is possible that, in the not far away future, patients with specific pathogenic variants may be treated.

### Cost-effectiveness of the gene panel

Although the estimated cost per sample for the gene panel (R$ 800.00) is higher when compared with that of IFM (R$ 500.00), it is considerably lower than that of TEM (US$ 980.00; R$ 3,920.00). However, it is important to note that the cost of TEM was based on prices charged in laboratories in the United States, since, to the best of the authors’ knowledge, the method is not offered commercially in Brazil. Although some Brazilian universities have an electron microscope, the method used to diagnose the disease is used in these academic centers only, for cases of specific scientific interest. Sanger sequencing has a mean price of R$ 35.00 per analyzed gene fragment (usually an exon), per sample. Thus, the final value of this methodology depends on the number of exons of the analyzed genes. Hypothetically, if an individual with dystrophic EB (associated with the *COL7A1* gene) was analyzed by Sanger sequencing, a value of approximately R$ 2,520.00 would be invested with this methodology alone (taking into account a number of 72 pairs of primers that include the 118 exons of the gene, as described by Christiano et al.^22^).

Thus, although the gene panel is not a low-cost method, the result obtained from it is more informative and conclusive than that obtained by IFM and TEM. In comparison to Sanger sequencing, the cost is lower, as well as the processing time, showing the cost-effectiveness of the gene panel for the diagnosis of EB. In addition, the price of this methodology has been decreasing over time, which should make the method more accessible in the coming years.^10^

It is worth mentioning, however, that the estimated cost in this study for diagnosis through the gene panel did not include the panel purchase price (approximately R$ 20,000.00) and the investment to validate it. The present proposal is that genetic diagnosis is provided in the context of public health, aiming, in the long term, to include the largest number of patients with EB in Brazil. Thus, as more patients are analyzed, the cost invested in the panel, per patient, decreases to the point of being negligible. In addition, the cost to be invested in an analyst with expertise to assess the results was not added to the calculation. In the authors’ experience, in an academic context, this question doesn’t apply; however, it should be taken into account before providing the method in the scope of public assistance.

### Brazilian panorama: clinical examination does not solve all cases of EB

The diagnosis of EB in Brazil is made, in most cases, through the evaluation of the clinical signs presented. There are few specialized centers that offer EB diagnostic methods, which results in difficulties in diagnosing and subclassifying patients and, consequently, providing correct genetic monitoring and counseling.

Even when available, IFM often results in inconclusive results, due to the lack of experienced analysts, especially in poorer and more remote Brazilian regions. Taking into account the damage caused by skin biopsy exams and the fact that most patients do not have the appropriate care after the exam (including appropriate dressings and healthcare), some physicians choose not to indicate IFM to subclassify EB. Consequently, the precise diagnosis and definition of the type of EB rely on a complete clinical examination. However, even when performed by dermatologists specialized in EB, clinical examination is not sufficient to solve all cases, in view of the extreme phenotypic variability observed. Table 2 presents an example of a clinical case whose genetic result did not coincide with the previous clinical hypothesis, surprising dermatologists. Similar cases have been described in previous studies and indicated that the effect of genetic variants may result in atypical clinical phenotypes.^23^, ^24^

**Table 2:** Clinical examination does not solve all cases of EB.

Clinical case
A 32-year-old patient was diagnosed late with epidermolysis bullosa (at the age of 15 years) due to the mild clinical presentation. The patient's symptoms were subtle when compared with more severe cases of EB. Blisters were distributed in the pre-tibial region and only a slight presence of blisters was found in the hands. Nail dystrophy was observed only on toenails. The patient underwent a skin biopsy which was inconclusive. The clinical examination carried out by expert dermatologists suggested the probable type of EB: localized EB simplex. The genetic analysis by the gene panel was in contrast to what was expected by the clinicians: two pathogenic variants were found in the *COL7A1* gene, revealing that it was a case of recessive dystrophic EB (RDEB). The most well-known phenotype of RDEB is quite severe, involving high induction and generalized distribution of blisters and pseudosyndactyly. Mild forms of RDEB are more rare and difficult to determine by clinical examination. Studies have shown that genetic alteration has a great influence on clinical presentation. Both pathogenic variants identified in the patient act by altering the splicing of *COL7A1*. Other very mild cases of RDEB have already been associated with this same type of variant. The case reported here exemplifies the importance of genetic analysis for the correct determination of the type of EB. Since the pattern of inheritance varies between types of EB (EBS: dominant, JEB: recessive, DEB: dominant or recessive), knowledge of its classification is essential for correct genetic counseling.

In the authors’ experience in Brazil, in most cases an accurate diagnosis of the type of associated EB was only possible after the genetic results, which in turn allowed for correct genetic counseling. In addition, some peculiarities of the country are also important. The rates of endogamy and consanguinity are higher in some Brazilian populations, especially in the Northeast, suggesting a high prevalence of recessive genetic inheritance.^25^, ^26^ In fact, in a previous study, the authors found that 82.7% of Brazilian patients with DEB subtypes had recessive inheritance.^13^ Determining the pattern of inheritance is especially important for the diagnosis of localized DEB subtypes, since the dominant and recessive forms are clinically indistinguishable, hindering correct counseling in the absence of genetic testing.

Considering the difficulty of making an accurate clinical diagnosis and, particularly, the complexity of classifying EB, genetic results proved to be essential for the correct determination of the type of associated EB.

### Gene panel as the first option for the diagnosis of EB

The survey carried out in this study demonstrated that gene panel is an effective tool for the conclusive diagnosis of EB, with high efficiency, diagnostic precision, and reduced analysis time (approximately ten days), being economically viable and waiving the need for invasive tests (Fig. 1). Some factors, however, are paramount for the effectiveness of the method. Processing the results of the gene panel requires support from bioinformatics, and the time taken to analyze the variants depends on the researcher's experience. However, when compared with the methods of IFM and TEM, the interpretation of the results of the gene panel is simpler, not requiring a team with extensive expertise.^3^

In light of the greater diagnostic efficiency, the robustness of the results obtained, the price comparable to the techniques currently adopted, and the possibility of genetic counseling, prognosis, and prenatal diagnosis, the present study supports the use of the gene panel as the first choice for the diagnosis of EB in Brazil. Taking into account the continental dimensions of the Brazilian territory and the difficulty of introducing a detailed technique such as the gene panel, the establishment of a reference center for the diagnosis of EB in the country would bring many benefits. The centralization of analyses in a single place eliminates the need to create multiple specialized teams and reduces the cost of processing per sample.

The difficulty of diagnosing patients with EB in Brazil can be mitigated with the methodological approach proposed in the present study. For this plan to become a reality, financial resources and government support are needed. It is essential that the authorities pay attention to this group of patients who have a serious genetic disease and who, although rare, represent a group of more than 900 Brazilians.

## Conclusions

The EB gene panel has a high diagnostic efficiency and eliminates the need for biopsy, in contrast to the methods currently adopted. The cost per patient of the presented methodology is comparable to that of the other techniques and will tend to decrease in the coming years. The detection of variants associated with EB reveals the prognosis of the disease, allows for prenatal and pre-implantation genetic diagnosis, and has implications for genetic counseling. Finally, although still in preclinical stages, potential therapies for EB have shown promising results for some specific types of genetic variants. The present results support the gene panel combined with clinical examination as the first choice for the diagnosis of EB in Brazil.

## Financial support

This study received financial support from the National Institute of Science and Technology of Population Medical Genetics (INaGeMP/CNPq/FAPERGS), number 465549/2014-4, and from the Coordination for the Improvement of Higher Education Personnel (10.13039/501100002322CAPES).

## Authors’ contributions

Luiza Monteavaro Mariath: Drafting and editing of the manuscript; critical review of the literature, design and planning of the study; genetic and bioinformatic analysis.

Ana Elisa Kiszewski: Approval of the final version of the manuscript; critical review of the manuscript; design and planning of the study; patient recruitment; dermatological assessment of patients.

Jeanine Aparecida Frantz: Approval of the final version of the manuscript; critical review of the manuscript; patient recruitment; dermatological assessment of patients.

Marina Siebert: Approval of the final version of the manuscript; critical review of the manuscript; genetic assessments; cost-effectiveness assessment of the gene panel.

Ursula Matte: Approval of the final version of the manuscript; critical review of the manuscript; genetic assessments; cost-effectiveness assessment of the gene panel.

Lavínia Schuler-Faccini: Approval of the final version of the manuscript, conception and planning of the study, critical review of the manuscript; literature review.

## Conflicts of interest

None declared.
